# Aging and Wound Healing of the Skin: A Review of Clinical and Pathophysiological Hallmarks

**DOI:** 10.3390/life12122142

**Published:** 2022-12-19

**Authors:** Kamarul Ariffin Khalid, Ahmad Faris Mohd Nawi, Nuraqilah Zulkifli, Md. Abul Barkat, Hazrina Hadi

**Affiliations:** 1 Department of Orthopaedics, Traumatology & Rehabilitation, Kulliyyah of Medicine, Kuantan 25200, Malaysia; 2 Department of Orthopaedics, Traumatology & Rehabilitation, Sultan Ahmad Shah Medical Centre (SASMEC) @IIUM, Kuantan 25200, Malaysia; 3 Dermatopharmaceutics Research Group, Kulliyyah of Pharmacy, International Islamic University Malaysia, Kuantan 25200, Malaysia; 4 Department of Pharmaceutics, College of Pharmacy, University of Hafr Al-Batin, Hafr Al Batin 39524, Saudi Arabia; 5 iKOP Pharma Sdn. Bhd., International Islamic University Malaysia, Kuantan 25200, Malaysia

**Keywords:** intrinsic aging, extrinsic aging, wound healing, aged skin

## Abstract

Aging is a universal process that can cause diminished function of organs and various diseases. The most striking consequences of aging can be seen visibly on the skin, which acts as a barrier against various external insults. Aging of the skin consists of intrinsic and extrinsic processes that work in concert and influence each other. Intrinsic aging involves biochemical degenerative processes that gradually takes place with age. Extrinsic aging are biochemical processes driven by external influences that lead to aging. There are significant morphological changes at all levels in aged skin that have a profound effect on the characteristics of the skin. Even though skin is subjected to damage by external insults, it is equipped with a healing capability in order to restore its normal structure and function. However, aging has a significant impact on the skin’s healing function by prolonging the inflammatory phase and increasing the production of reactive oxygen species (ROS). This shifts the healing process towards having more protein degradation, which can lead to chronic wound healing with an abundance of complications.

## 1. Introduction

Human civilization has overcome countless obstacles during its long history, mainly through the advancement of knowledge and technology. However, despite all the accomplishments, there are still some boundaries that we have yet to cross, despite our best efforts. One of them is aging. Aging is a universal process that we, as human beings, are incapable of slowing down, let alone stopping [1,2]. No one is exempted from it and, with time, everyone will end up with grey hair, weak joints, wrinkles, and sagging skin or, eventually, might not even be able to recall their own name. Thus, it is easy to understand why aging is a major concern for everyone.

Aging is associated with declining health, as it is regarded as a risk factor for most medical conditions, such as cardiovascular diseases, cancers, and physical limitations [3,4]. From a cosmetic point of view, aging also affects the skin’s normal physiological function, as it will not be functioning the same way when aged. For instance, wound healing, which is a normal physiological response, occurs at a slower rate in aged skin and can usually end up with complications [5]. This has led to an increased effort to study the mechanism of skin aging and how it is related to wound healing. Even though aging is inevitable, with proper knowledge of skin aging, it may be possible to delay its effects with the help of technological advancements. Thus, in this paper, we will be discussing how skin aging occurs and how it can affect the process of wound healing.

## 2. Prevalence of the Aging Population

Old age is known as the sunset of life, as the body does not function in the same way that it did when younger. The specific age classified as elderly in the third world and developing countries is 60 years old, whereas in the developed countries it is 65 years old [6]. In 2019, global life expectancy increased to 73.4 years from 66.8 years in 2000 [7]. This is due to the massive improvements in hygiene practice and medical advancement and also increased food supply. The increasing life expectancy has resulted in an aging population, which is currently a worldwide phenomenon [8]. Japan has the highest percentage of elderly in their population at 28.2%; their population distribution has shifted towards advanced age rather than newborns due to this, as well as reduced fertility [9]. This is a cause for concern as aging population leads to economic stunting due to shortage of labour and increased cost of healthcare services. Malaysia is expected to achieve an aging population status by 2035, when 15% of the total population exceeds 60 years old [2].

Even though the increase in life expectancy is very much welcomed by everyone, it can also be considered as an emerging problem. This is because the elderly are usually highly dependent as they are more likely to be subjected to age-related diseases. Other than that, the skin will also undergo changes in parallel with age. The majority of people aged more than 65 years usually suffer from at least one skin disorder such as xerosis, malignancy, and skin injuries [10]. Thus, it is important to understand how aging affects the normal physiology of skin.

## 3. Mechanism of Skin Aging

Skin is an organ that covers the entire body and is considered the human’s largest organ with a total surface area of 2 m^2^ and weight of 3.6 kg [11]. It consists of three distinctive layers, which are the epidermis, dermis, and hypodermis. Its main function is to act as a barrier that provides protection against mechanical stress, extreme temperatures, harsh toxins, radiation, and microbes [12,13,14]. Eventually, upon continuous exposure towards external as well as internal insults, skin will undergo modification to its morphology and function, which leads to symptoms of aging [13]. Not only that, it will also contribute to numerous age-related deficits such as skin malignancy and defective wound healing. In short, skin is subjected to both intrinsic and extrinsic aging, and both of these process works in concert and influence each other.

### 3.1. Intrinsic Aging

Intrinsic aging is also known as chronological aging. It is a biochemical degenerative process that takes place gradually as a result of growing older. The intrinsic aging of skin is mainly characterized by unblemished, smooth, pale, and fine wrinkles with less elasticity and prominent dryness [15,16]. It is often seen in the elderly, with genetic and hormonal factors as the main triggers that drive the process. Thus, a significant variation can be found not only between differing populations and individuals but also within the same ethnicity and different body locations within an individual [13]. Stress hormone cortisol is a major driver of skin aging. The high amount of cortisol in aging is related to increasing psychosocial stress, decreased cognitive performance, and atrophy of memory-related composition in the brain, like the hippocampus [17]. This high cortisol level can negatively affect some of the extracellular matrix proteins by breaking down collagen, proteoglycans, and elastin [18]. DNA damage and telomere shortening are found to be the major factors that lead to the defect in cell proliferation capacity [16].

Day-to-day external insults, such as radiation and chemical exposure, may cause damage to DNA, but humans are equipped with a powerful DNA repair mechanism that is able to offset the damage [19]. However, as the age increases, the repair mechanism efficiency is reduced, which causes some DNA errors to escape the repair mechanism. Thus, this increases the likelihood of older cells to contain DNA errors which result in genetic instability. Subsequently, accumulated damaged DNA may lead to carcinogenesis and other age-related disorders [20].

Telomere shortening is also another major cause for most age-related diseases and premature aging [21]. The telomere is a region located at each end of the chromosome that comprises a repetitive nucleotide sequence that protects the ends of the chromosome and maintains the genomic stability. Humans are incapable of extending the telomere, as human somatic cells do not express telomerase; an enzyme with the role of extending the telomere end of DNA [13]. Thus, with each cell division, the DNA strands will gradually shorten and eventually reach the DNA coding region. At this stage, the cell will enter replicative senescence known as the “Hayflick limit”, which limits its replicative capacity [22]. As skin loses its replicative capacity, there are major alterations in its structure and functions.

### 3.2. Extrinsic Aging

On the other hand, extrinsic aging involves biochemical processes driven by external influences instead of the individual’s genetic makeup. Extrinsic aging is usually characterized by deep wrinkles, rough texture, dullness, patchy hyperpigmentation, and loss of skin elasticity [15]. It occurs at a more rapid pace and can also be seen in younger people. Extrinsic aging is mostly triggered by external factors such as environmental (ultraviolet radiation (UVR), air pollution), mechanical (muscle strain that leads to tissue plasticity), and lifestyle (diet, sleep pattern, cigarette smoking) changes [13,16]. By far, continuous unprotected sun exposure can be considered as the major contributor that leads to extrinsic aging. This type of aging is known as photoaging. Usually, it is prominent throughout exposed areas such as the face, neck, hands, and legs. Not only that, about 80% of facial skin aging is caused by continuous exposure towards UVR [15,16]. The degree of sun exposure will determine the magnitude and extent of skin aging. Other than that, cigarette smoking was also found to accelerate skin aging, which mainly targets elastin in the skin [23]. The reduction in elastin will lead to loss of tissue elasticity and, consequently, results in a distinct pattern of prominent perioral lines and sharply contoured crow’s feet usually cited as the “smoker’s face”.

## 4. Observation on Skin Aging

### 4.1. Epidermis

The outermost layer of the skin is known as the epidermis. It is 15 to 40 times thinner as compared to the dermis [13]. It consists of keratinized stratified squamous epithelium that is not vascularized. The components of the epidermis can be categorized into two parts. The first part is known as the stratum corneum, which lines the outermost section of the skin. It consists of dead flattened corneocytes that serve as the main barrier towards external insults as well as maintaining optimal skin hydration. The second part mainly comprises viable cells such as keratinocytes (90–95%), Langerhans cell (2%), melanocytes (3%), and Merkel cells (0.5%) [15]. At the deepest part of the epidermis is the basal lamina, which comprises the epidermal stem cells. These stem cells contribute to its high proliferative capacity. It is able to differentiate into keratinocytes that will then migrate towards the stratum corneum through the keratinization process [13,24]. It will push the older keratinocytes towards the surface and eventually become flattened corneocytes and die. However, the rate of epidermal cell turnover significantly reduces by half within the age of 30 to 70 [13].

The effect of aging in the epidermis is controlled by the balance between extrinsic and intrinsic factors on the skin. The most striking changes that affect the epidermis can be seen in the basal lamina, which becomes thinner. Intrinsic aged skin often exhibit diminished cell proliferative capacity at the basal lamina [25]. This is because the basal keratinocytes down-regulate the expression of β1-integrins, which leads to abnormal proliferation and adhesion [15]. Consequently, keratinocytes will become shorter and flatter, whereas the corneocyte will increase in size with age. Conversely, the stratum corneum is mostly affected by UVR. It usually thickens as the corneocyte desmosome, a cell structure specialized for cell-to-cell adhesion, fails to degrade completely [25]. This interferes with the normal cellular shedding and, hence, reduces the epidermal cell turnover. In conjunction to that, aging also compromises the barrier function and recovery of the skin [15]. This will cause increased transepidermal water loss that will reduce the skin hydration and lead to xerosis. The sum effect of the increased thickness of stratum corneum and reduced epidermal water content will result in the increased stiffness of the skin [13].

### 4.2. Dermal-Epidermal Junction (DEJ)

Dermal-epidermal junction (DEJ) is located exactly below the epidermis and serves as the border between the epidermis and dermis [26,27]. DEJ basically provides an interface for nutrition exchange, cellular signaling, and waste removal for the avascular epidermis. With increased age, the DEJ will flatten out due to reduced papillae density [13,28,29]. This will lead to a reduction (up to 80%) in the epidermal thickness. Other than that, exposure to UVR also accelerates the rate of DEJ thinning as it alters type IV collagen, which is the main constituents of the DEJ [15]. The receding DEJ will reduce the surface area of contact between epidermis and dermis, which eventually reduces nutrients and waste exchange. As a consequence, the basal proliferation will be inflicted with not only reduced nutrient supply but also waste accumulation that leads to reactive oxygen species build up [25]. Moreover, the flattening of DEJ weakens the connection between epidermis and dermis, which reduces its resistance towards shearing force and leads to wrinkle formation [15,29].

### 4.3. Dermis

The middle layer of the skin is known as dermis. It contains an abundance of connective tissues and skin appendages such as sweat glands, sebaceous gland, blood vessels, and nerves. The main role of the dermis is to act as the main tensile bearing component of skin, which gives the skin its mechanical strength [13]. Specifically, there are two distinctive regions of the dermis, which are the superficial papillary dermis and deeper reticular dermis. The superficial papillary dermis is also defined as rete ridges, which are finger-like projections that extend into the epidermis and mainly comprised loose connective tissue. This structure enables it to supply nutrients and take up waste from the avascular epidermis. In contrast, the deeper reticular dermis comprises dense connective tissue that makes up the bulk of the dermis [30]. Other than that, fibroblasts can also be seen in the dermis layer. It produces materials for the extracellular matrix (ECM), such as collagen, elastin, and glycosaminoglycan, which also contributes to the skin bulk, strength, and resilience. Fibroblasts are often found to form adhesion complexes within the dermal ECM, which allows it to spread and exert mechanical force throughout the dermis [31].

Collagen, the most abundant protein found in the skin, contributes towards the skin tensile strength [11,32]. Alteration of the skin collagen has an impact on skin aging. In young skin, the collagen meshwork is regularly organized and tightly packed within the papillary dermis [13]. It also spreads into the epidermis forming DEJ, which further provides tensile support to the skin. Not only that, the major constituent of collagen in young skin is type I collagen (80%) as opposed to type III collagen (15%) [15,24]. However, in aged skin, the dermis is severely damaged and exhibits thinning, which is mainly due to increased collagen fragmentation and reduction of type I collagen in the dermis.

In aged skin, there is also an increased expression of matrix metalloproteinase (MMPs), which is a degradative enzyme of collagen fibrils, and leads to its fragmentation [13,33]. Consequently, there will be an increased rate of collagen cleavage leading to the accumulation of fragmented cross-linked collagen that will eventually cause the ECM to be degraded and disorganized. In addition, the fragmentation of collagen also leads to the extrinsic aged skin appearance, such as wrinkles and fragile atrophic skin [34]. The collagen fragmentation also causes loss of site for fibroblast attachment [31,33], resulting in reduced fibroblast spreading [31]. Thus, there will be lesser fibroblast available to produce new collagen fibers. Aged skin also exhibits an increase in the enzyme cyclooxygenase-2 (COX-2), important in arachidonic acid metabolism [35], and one of its by-product is prostaglandin E2 (PGE2), which inhibits collagen production by fibroblasts. Thus, it can be concluded that collagen lost in aged skin is mostly due to the imbalance between collagen synthesis and degradation.

The next Important connective tissue of the ECM is elastin. Elastin mainly contributes towards skin elasticity and resilience, as it is closely intertwined with the collagen fibers [36,37]. Any distinctive loss of skin elasticity mostly happens due to abnormalities in elastin production. One of the main insults includes UVR, which triggers the remodeling and degradation of elastic fibers via the activation of MMPs [15]. Apart from that, UVR also induces splicing of the elastin gene, leading to a reduction in elastin synthesis. Subsequently, a reduction in elastin as well as collagen will result in a stiffer and more rigid dermis. This will lead to the chronic cutaneous fragility condition known as dermatoporosis [24]. Dermatoporosis is often associated with prominent features such as skin atrophy, solar purpura, and a whitish scar-like formation on the skin. Wound healing is also found to be decelerated in dermatoporosis, causing vulnerability to infection and bleeding [38,39].

Glycosaminoglycans and proteoglycans are also abundantly found in ECM. One of the most important glycosaminoglycan in skin is hyaluronic acid, which is a non-sulphated form of glycosaminoglycan [40]. Hyaluronic acid is considered as a principal molecule for skin moisture. This is mainly due to its capacity to hold water molecules up to 1000 times its molecular weight [41]. As it is extremely hydrophilic with strong water binding ability, hyaluronic acid is responsible for maintaining the skin moisture state and making it pliable. About 50% of hyaluronic acid can be found specifically in the skin [42]. This causes young skin to be well hydrated, as it is rich in hyaluronic acid [15]. However, there are dramatic changes in the hyaluronic acid found in aged skin; there is a reduction in both epidermal and dermal hyaluronic acid [42]. Hyaluronic acid is synthesized by fibroblasts [41], but with the reduction in fibroblast spread due to the increased fragmentation of collagen in aged skin, as previously explained, there are less fibroblasts available for its production. The depletion of hyaluronic acid leads to the impairment of water retention ability causing loss of skin moisture. In addition to that, the presence of hyaluronic acid also ensures the proper configuration of collagen and elastin to remain intact [40]. Thus, the reduction in hyaluronic acid and increased collagen and elastin disorganization in aged skin leads to the prominence of fine lines, wrinkles, and nasolabial folds.

**Figure 1:**
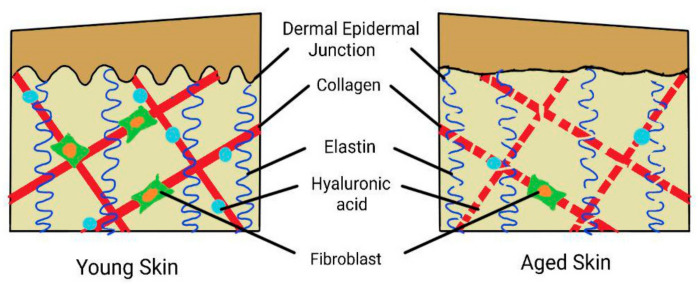
Summary of the effects of aging on the skin. The dermal epidermal junction (DEJ) is flattened, and in the dermis is a reduced number of fibroblasts with fragmentation of collagen, elastin reduction, and depletion of hyaluronic acid.

Proteoglycan is a family of glycosaminoglycan that is covalently bonded to one or multiple glycoprotein chain. The roles of proteoglycan include maintaining the skin’s hydration state, imparting skin resistance and resilience, involvement in molecular filtration, and controlling cell-to-cell interactions [43]. The most abundant proteoglycans that can be found in the skin are decorin, biglycan, and versican [41]. Decorin helps in assembling collagen fibrils into compact fibres, as its glycosaminoglycan chain is able to control the collagen fibre diameter [44]. However, different expressions of decorin can be found in photo-aged and chronologically aged skin. In photo-aged skin, there is a significant reduction of decorin. This is due to the increased fragmentation of collagen that reduces the available sites for decorin binding. In chronologically aged skin, on the other hand, there is an accumulation of decorin but in much smaller amounts as compared to young skin [43]. In conjunction to that, decorin also possesses a growth inhibitory effect that leads to cellular senescence and explains the increased production of decorin in aged skin. Biglycan deficiency, though, will lead to abnormal synthesis of collagen fibril, resulting in dermal thinning [45]. Moreover, versican contributes to skin tensile strength as well as maintaining the skin’s hydration state [15]. The expression of versican in aged skin is mainly reduced, but there is an accumulation of versican in photo-aged skin. This is due to UVR exposure, which up-regulates the expression of versican [43]. The accumulation of versican will initiate a negative feedback mechanism that reduces the synthesis of versican. Thus, this will be followed by loss of versican in the dermis, which contributes to the loss of skin elasticity, resilience, and moisture. The summary of the effects of aging is illustrated in Figure 1.

## 5. The Effects of Skin Aging on Wound Healing

### 5.1. Normal Wound Healing Physiology

Skin, which comprise epithelial cells, has the ability to regenerate throughout life [46,47]. This high proliferative capacity is important for skin due to its main principle function, which is to act as a barrier against external and internal insults [46,48]. Thus, it needs to be constantly replaced with new epithelial cells once it had been damaged by those insults. Skin is equipped with an innate immune response towards tissue injury, with the aim to restore normal tissue structure and function [46]. The normal wound healing process comprises three distinctive stages, inflammation, reepithelialization, and tissue remodelling [32].

#### 5.1.1. Inflammatory Phase

Immediately after tissue injury, there is a transient vasoconstriction of the injured micro-vessels. This helps to reduce the blood flow and increases the contact time for clotting factors to make contact with the injured site to form a primary plug. When the primary plug has fully formed, it will stop the bleeding and reduce further blood loss. At the same time, the primary plug and the surrounding cells also release a cocktail of cytokines, chemokines, and growth factors, such as platelet-derived growth factor (PDGF), epidermal growth factor, vascular endothelial cell growth factor (VEGF), and transforming growth factor β (TGF-β), which promote the migration of inflammatory cells to the site of injury [49,50]. Within the first 24 h after tissue injury, acute inflammation takes place, whereby neutrophils predominate at the injury site [51,52]. The neutrophils phagocytose any invading microorganisms as well as remove tissue debris. Then, by 24 to 48 h, monocytes migrate to the site of injury [52,53,54] and become activated into macrophages. Depending on the function, macrophages can be classically activated (M1), which exerts a pro-inflammatory function, or alternatively activated (M2), which promotes wound healing [53]. At first, M1 predominates, which helps in eliminating apoptotic cells and spent neutrophils, and also fights against infection. Then, M2 takes over the role to mediate tissue repair by secreting cytokines and growth factors. Other than that, T cells are also involved in the healing process, whereby they interact with macrophages, platelets, leukocytes, and fibroblast to initiate tissue repair [49].

#### 5.1.2. Reepithelialization Phase

Once the inflammation phase has subsided, the wound healing process continues with reepithelialization. The major event that occurs at this phase involves keratinocytes proliferation and migration [46,52,54]. The injury site will be covered by the proliferation of adjacent cells and maturation of stem cells to restore the original skin structure and function. At day 3 (72 h), there is a formation of granulation tissue, which comprises fibroblasts, inflammatory cells, and new blood vessels, filling the injury site that can last up to 14 days [49,52]. The secretion of growth hormones such as TGF-β1, PDGF, and fibroblast growth factor (FGF) stimulate fibroblasts to proliferate and synthesize the ECM constituents, such as collagen, elastin, and glycosaminoglycan. During the initial synthesis of collagen, it is mostly oriented vertically and does not possess strong tensile strength. At the same time, angiogenesis also takes place in order to supply the extra nutrients and oxygen to support the tissue repair [51].

#### 5.1.3. Tissue Remodeling Phase

**Figure 2:**
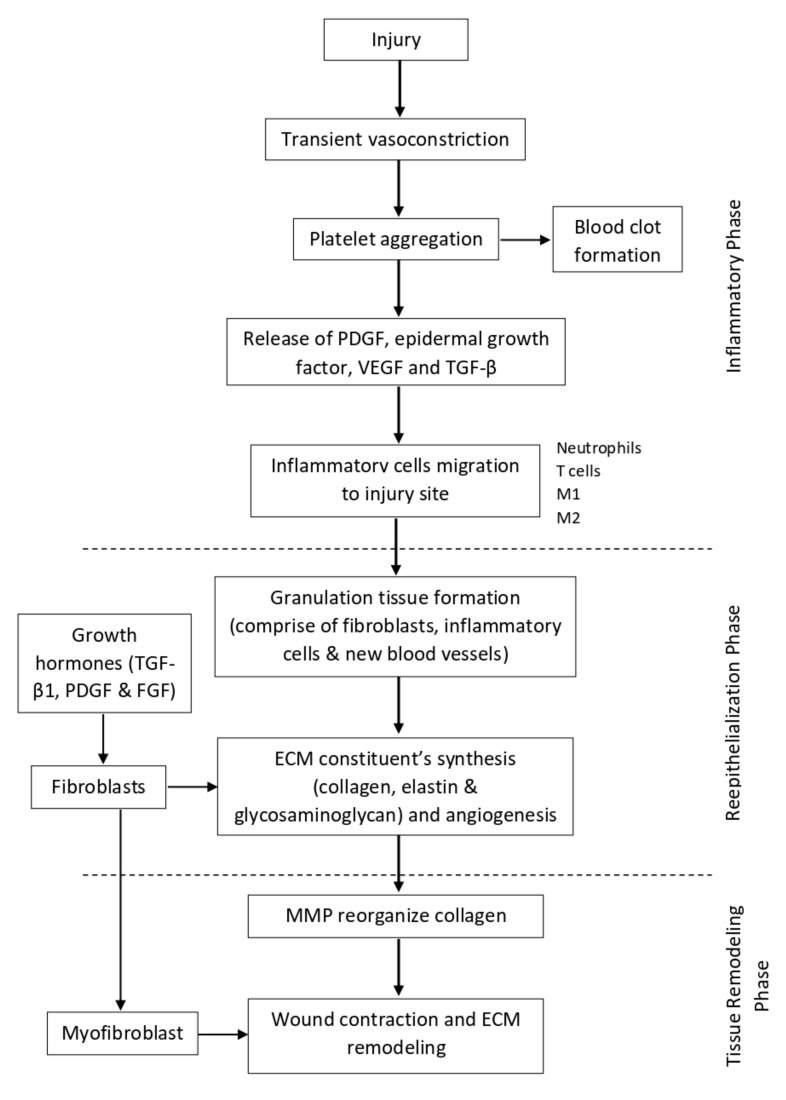
Illustration of normal wound healing physiology.

The last stage of wound healing is known as the tissue remodeling phase. It usually starts at day 8 and can persist up until 1 year or even longer [53]. At this stage, the ECM proteins are continuously degraded and synthesized to achieve the normal tissue architecture. One of the key enzymes that mediates this stage is MMP. It helps to reorganize the collagen bundles from vertically oriented into a horizontal orientation so that it can be more stable and exert a stronger tensile strength. The ratio of type III collagen also gradually reduces and is replaced with stronger type I collagen [32,49,52]. Other than that, some of the fibroblasts also differentiate into myofibroblast, which is involved in wound contraction and ECM remodeling [50,54,55]. By the end of the stage, the skin tensile strength can be restored up until 70–80% of normal skin, but it will never reach its full potential as before. A diagram illustrating the normal wound healing physiology can be seen in Figure 2.

### 5.2. Alteration of Wound Healing Process Due to Aging

#### 5.2.1. Prolong Inflammatory Phase

The balance between inflammation and its control is essential to maintain a normal wound healing process [56]. This is because acute inflammation at the early stage of wound healing is beneficial in removing cell debris and invading microbes. However, if the inflammation state is prolonged, this will lead to further destruction of adjacent cells and eventually inhibit wound healing. Aging causes more platelets to adhere to the injured epithelium. This will cause the production of more pro-inflammatory cytokines such as PDGF, TGF-β, and TGF-α [57]. In response to their release, neutrophils will rapidly be recruited to the site of injury. Simultaneously, monocytes will also be recruited to the site of injury. However, since monocytes are larger in size, they require specific adhesion molecules such as ICAM-1 and VCAM-1 to be expressed on the endothelial surface in order to infiltrate the site of injury [46,58]. In aged skin, these adhesion molecules are greatly reduced, and this impairs the monocyte infiltration [46,59]. Thus, there will be a lesser amount of monocytes being able to infiltrate and mature into macrophages at the site of injury.

Macrophage depletion causes impairment of the wound healing process by reducing granulation tissue formation and angiogenesis as well as impairment of collagen and growth factor synthesis [46,60]. There are also less macrophages being alternatively activated, which is crucial for the transition from the inflammatory phase to the proliferative phase [23]. Once the macrophages are alternatively activated, it will produce IL-10 and reduce the production of IL-12. This will halt inflammation and initiate tissue repair. At the same time, tissue injury will also increase the production of pro-inflammatory cytokines that activates the COX pathway [56]. This will cause an increase in the production of PGE2. It will induce the inflammatory response that further degrades the adjacent cells and delays the healing process. Not only that, PGE2 also have an inhibitory role on fibroblasts [35]. This inhibits collagen synthesis by fibroblast and impairs the proliferative phase. T cell infiltration is also delayed in aged skin, but the effects are still unknown [46].

#### 5.2.2. Oxidative Stress

Reactive oxygen species (ROS) also have an important role in the wound healing process. They are produced by neutrophils and macrophages via NADPH oxidase and help in killing microbes and preventing wound infection [61]. They also have a role as a vasoconstrictor to help reduce blood flow and promote thrombus formation soon after injury [49]. Oxidative stress is also necessary for transition into the proliferative phase [5]. A low amount of ROS provides positive effects on wound healing, whereas prolonged exposure to ROS has a detrimental effect on the wound healing process. Increased production of ROS, such as nitric oxide and superoxide anions, will increase tissue damage and impair healing [23]. In aged skin, more ROS will accumulate due to prolonged inflammation. Hence, this will prolong the state of oxidative stress [62,63]. A prolonged state of oxidative stress will also increase lipid peroxidation and promote protein breakdown and DNA damage, which, in turn, will impair wound healing through increased cell apoptosis and senescence [64,65]. This will significantly impair the proliferative phase and prolong the wound healing process.

#### 5.2.3. Inefficient Microcirculation

Aged skin will usually have damaged and impaired growth of blood vessels [60,64]. Once the microcirculation is impaired, there are changes in the inflammatory response due to a reduction in the inflammatory cells and chemical mediators being able to reach the site of injury [5]. On top of that, it also means that there is a relative hypoperfusion at the injury site, leading to less nutrients and oxygen being able to be supplied to support the wound healing process. This impaired blood supply can also lead to hypoxia, which causes cell death due to apoptosis and necrosis [64]. Temporary hypoxia is important in wound healing process as it can stimulate the release of cytokines and growth factors to induce cell proliferation, migration, as well as angiogenesis [46]. However, a prolonged hypoxia state will negatively affect the wound healing process. A summary of the effects of aging on wound healing can be seen in Table 1.

## 6. Conclusions

Although the principal role of skin is to protect us against both external and internal insult, it does not mean that these insults do not have degrading effects on the skin. The skin is subjected to both intrinsic and extrinsic aging, whereby the former cannot be completely avoided at this time, whereas the latter can be avoided through proper care such as wearing a sunscreen, maintaining a good diet, and practicing a healthy lifestyle. Appropriate measures need to be taken to protect and shield our own skin from these insults. Continuous exposure to the insults will alter skin function and can lead to numerous deleterious effects in the future. Ultimately, aging skin will have distinctive changes in appearance such as deep wrinkles, hyperpigmentation, loss of elasticity, and prominent dryness. Impairment of wound healing is also linked with the aging process of skin, leading to a prolonged and impaired wound healing process. Thus, instead of the skin healing rapidly, it can progress a to chronic state that will increase the susceptibility of having wound infection and scarring. The identification of the molecular pathways to intrinsic skin aging is a key factor in finding ways to prevent it and the negative effects it has on wound healing for the future.

## Figures and Tables

**Table 1:** The effect of skin aging on the wound healing process.

Effect of Aging on Wound Healing	Cell/Tissue/Organ Involved	Action	References
Prolong inflammatory phase	↑Platelets	↑Pro-inflammatory cytokines (e.g., PDGF, TGF-β & TGF-α)	[57]
	↓Adhesion molecules	Impairs the monocyte infiltration	[46]
	↓Macrophage	↓Granulation tissue formation, angiogenesis, collagen and growth factor synthesis ↓M2, hence prolong inflammation and halt tissue repair	[46,56]
	↑Pro-inflammatory cytokines	Activates the COX pathway ↑PGE2 production	[35,56]
	↑PGE2	↓Fibroblast and collagen synthesis, hence impairs the proliferative phase	[35]
Oxidative stress	↑Reactive oxygen species (ROS)	↑Tissue damage, lipid peroxidation, promote protein breakdown and DNA damage, hence↑cell apoptosis and senescence	[23,64]
Inefficient microcirculation	Impaired blood vessels	↓Inflammatory cells and chemical mediators at injury site Relative hypoperfusion at injury site Hypoxia that causes cell death due to apoptosis and necrosis	[5,64]

↑ Increase. ↓ Decrease.

## Data Availability

Not applicable.
